# A Novel FEM Based T-S Fuzzy Particle Filtering for Bearings-Only Maneuvering Target Tracking

**DOI:** 10.3390/s19092208

**Published:** 2019-05-13

**Authors:** Xiaoli Wang, Liangqun Li, Weixin Xie

**Affiliations:** 1 Automatic Target Recognition (ATR) Key Laboratory, Shenzhen University, Shenzhen 518060, China; xlwang@szu.edu.cn (X.W.); wxxie@szu.edu.cn (W.X.); 2 Guangdong Key Laboratory of Intelligent Information Processing, Shenzhen University, Shenzhen 518060, China

**Keywords:** maneuvering target tracking, particle filtering, T-S fuzzy modeling, fuzzy expectation maximization

## Abstract

In this paper, we propose a novel fuzzy expectation maximization (FEM) based Takagi-Sugeno (T-S) fuzzy particle filtering (FEMTS-PF) algorithm for a passive sensor system. In order to incorporate target spatial-temporal information into particle filtering, we introduce a T-S fuzzy modeling algorithm, in which an improved FEM approach is proposed to adaptively identify the premise parameters, and the model probability is adjusted by the premise membership functions. In the proposed FEM, the fuzzy parameter is derived by the fuzzy C-regressive model clustering method based on entropy and spatial-temporal characteristics, which can avoid the subjective influence caused by the artificial setting of the initial value when compared to the traditional FEM. Furthermore, using the proposed T-S fuzzy model, the algorithm samples particles, which can effectively reduce the particle degradation phenomenon and the parallel filtering, can realize the real-time performance of the algorithm. Finally, the results of the proposed algorithm are evaluated and compared to several existing filtering algorithms through a series of Monte Carlo simulations. The simulation results demonstrate that the proposed algorithm is more precise, robust and that it even has a faster convergence rate than the interacting multiple model unscented Kalman filter (IMMUKF), interacting multiple model extended Kalman filter (IMMEKF) and interacting multiple model Rao-Blackwellized particle filter (IMMRBPF).

## 1. Introduction

The motion state (position, velocity, acceleration, etc.) estimation of the target in the system refers to the measurement information obtained by the measuring device, and establishes a reasonable and accurate dynamic model by using modern signal processing techniques such as a stochastic process, estimation and detection theory, and a filtering algorithm. When dealing with linear systems, the Kalman filter [1] theory is the optimal linear Bayesian estimation algorithm. Subsequently, some extended Kalman filters (EKF) [2,3] were proposed to further apply the Kalman filter (KF) theory to the nonlinear system. The basic idea of the EKF is to linearize the nonlinear system and then perform KF, so EKF is a suboptimal filter. Then, a second-order generalized Kalman filter [4] method was proposed and applied to further improve the estimation performance of KF for nonlinear systems. It took into account the second-order terms of the Taylor series expansion. Therefore, the estimation error caused by linearization was reduced, and the filtering precision of nonlinear systems was improved, but the computational complexity was greatly increased. Therefore, it was not widely used in practice. Otherwise, with the aggravation of the nonlinearity of the dynamic system, the performance of EKF decreases sharply. In order to solve this problem, Julier and Uhlmann proposed the unscented Kalman filter (UKF) [5] algorithm. Unlike the EKF, the UKF [6,7,8] approximates the distribution of state random variables by selecting a small number of sample points, using the nonlinear model directly. However, since EKF and UKF have no way of getting rid of the system’s Gaussian constraints, the processing effect on non-Gaussian systems is not good.

In order to deal with the problem of state estimation in the case of nonlinear and non-Gaussian systems, particle filtering (PF) [9] had been proposed. PF is a non-parametric Monte Carlo simulation method used to achieve a recursive Bayesian filtering. With the development of computing ability and statistical theory, the PF algorithm has been developed rapidly. Nowadays, the algorithm has been successfully applied in many fields. Isard et al. [10] introduced PF into the target tracking of image sequences, which made PF become a hot topic in the fields of target tracking, machine learning and robot localization. Then, scholars put forward many improved algorithms based on the PF algorithm, such as Unscented PF (UPF) [11], Rao-Blackwellized PF (RBPF) [12], Auxiliary PF (APF) [13], Regular PF (RPF) [14], MCMC PF [15] and Gaussian PF (GPF) [16,17].

Another significant feature in the nonlinear non-Gaussian system is the uncertainty of its motion pattern, so the traditional single-model method has difficulty achieving a good tracking performance. In view of this, some scholars have applied multiple PFs or a PF and multiple model method [18,19] to the system, and have obtained a good performance. Bando et al. [20] proposed a switching particle filter, which allowed robust and accurate visual tracking under typical circumstances of real-time visual tracking. This scheme switched two complementary sampling algorithms, condensation and auxiliary particle filter, in an on-line fashion based on the confidence of the filtered state of the visual target. Meshgi et al. [21] proposed an occlusion-aware particle filter framework that employs a probabilistic model, with a latent variable representing an occlusion flag, which prevented the loss of the target through the prediction of emerging occlusions, updated the target template by shifting relevant information, expanded the search area for an occluded target, and granted a quick recovery of the target after an occlusion. Martino et al. [22] introduced two novel Markov Chain Monte Carlo (MCMC) techniques based on group importance sampling, where the information contained in different sets of weighted samples was compressed by using only one (properly selected, however), particle, and one suitable weight. Otherwise, an interacting multiple model Bernoulli PF (IMMBPF) [23] algorithm for maneuvering target tracking was simply combined with the particle implementation of IMM and PF. The introduction of model information into the particle sampling process will lead to a reduction in the number of particles used to approach the current real state of the model, and the particles will interact with each other in each recursive model, which had the disadvantage of too much computation. To improve the effectiveness of single sampling particles in IMMBPF for real target states and a model approximation, Yang et al. [24] proposed an improved multiple model Bernoulli particle filter (MMBPF), in which the number of particles in each model was pre-selected. Furthermore, the particles in the model did not need to interact with each other, which reduced the computational load. The model probability was calculated from the model likelihood function, and the particle degradation of the small probability model was avoided without changing the Markov property of the model. Additionally, to meet the requirements of modern radar maneuvering target tracking systems and to remedy the defects of an interacting multiple model based on PF, a non-interacting multiple model (NIMM) and an enhanced particle swarm optimized particle filter (EPSO-PF) were proposed in [25]. NIMM was used to figure out the index of particles to avoid the high computing complexity resulting from particle interaction, and EPSO-PF not only improves the equation of a particle update through the rules through which individuals develop a group understanding but it also enhances particle diversity and accuracy through the small variation probability of the superior velocity. Additionally, the random assignment of an inferior velocity was capable of upgrading the filter efficiency. Instead of resorting to model selection, Urteaga et al. [26] fused the information from the considered models within the proposed SMC method, and achieved the goal by dynamically adjusting the resampling step according to the posterior predictive power of each model, which was updated sequentially as we observed more data. Martino et al. [27] designed an interacting parallel sequential Monte Carlo scheme for inference in state space models and in an online model selection. The parallel particle filters collaborated to provide a global efficient estimate of the hidden states and an approximation of the probability of the models, given the received measurements. For a static parameter estimation of the model, Carvalho et al. [28] extended existing particle methods by incorporating the estimation of static parameters via a fully-adapted filter that utilizes conditional sufficient statistics for parameters and states as particles. For the sake of a better tracking performance, it may be necessary to use a large set of models, but this will inevitably increase the computational complexity, which is one of the drawbacks of the multi-model filtering method. At the same time, unnecessary competition from too many models may result in a decline in performance. Therefore, it is of great value and practical significance to seek a more effective modeling approach.

As a mathematical tool to deal with the fuzzy phenomenon, fuzzy mathematics [29] can not only solve the uncertainty caused by the randomness of the system, but can also deal with the fuzzy uncertainty caused by the uncertainty of the extension of the concept of the system. It can be used to model qualitative, fuzzy or uncertain ones in the form of natural language. Fuzzy mathematics can describe different uncertain information in simple fuzzy language. Recently, the study of fuzzy particle filtering has become one of the research hotspots of complex nonlinear non-Gaussian systems. Widynski et al. [30] proposed a particle filtering algorithm with integrated fuzzy spatial information, which introduced the target spatial information through the fuzzy probability and improved the accuracy of the sampling particle. Li et al. [31,32] proposed a fuzzy orthogonal particle filter, which approximated the predicted probability density function and the posterior probability density function by introducing a set of positive intersection probabilities based on the Gauss-Hermite rule. The advantage of a fuzzy logic particle filter is that it does not need to know the statistical model of the process in advance. In addition, it does not need any maneuvering detector, even when tracking high-performance targets, so the computational complexity is low, and appropriate fuzzy overlapping sets will be closer to the real motion model. The theory of fuzzy models is a general concept proposed in the last decade. Among the various types of fuzzy models, there is a very important T-S [33,34] fuzzy model. Due to its special rules of consequence-structure and its success in function approximation, it has been widely used recently. Therefore, this paper constructs a general T-S fuzzy model framework based on spatial-temporal semantic information, and uses multiple linear models to get a more accurate target motion model, which makes the state estimation of the particle filtering algorithm more accurate.

In this paper, for the nonlinear non-Gaussian problem in the passive sensor system, a T-S fuzzy modeling particle filtering algorithm, based on improved fuzzy expectation maximization, is proposed. The main contributions are as follows: (1) A T-S fuzzy model, based on spatial-temporal information, is proposed for the uncertain modeling of a target dynamic model, in which spatial-temporal feature information is represented by multiple semantic fuzzy sets. Then, the general T-S fuzzy model framework is constructed, approximating the dynamic model with a high precision. (2) An improved fuzzy expectation maximization method with fuzzy C-regressive model clustering based on entropy and integrated spatial-temporal information is proposed for the premise parameter identification in the T-S fuzzy model. In addition, the model probability is adjusted adaptively through the premise membership functions. (3) The importance density function is constructed by using the proposed T-S fuzzy model, which contains abundant prior knowledge and the latest measurement information; thus, it can effectively approximate the true posterior probability density function and improve the diversity of particles.

The rest of this paper is organized as follows. Section 2 presents the proposed T-S fuzzy modeling particle filtering algorithm. Section 3 describes the simulation results that compare the performances of all of the algorithms. Finally, some conclusions of the proposed algorithm are given in Section 4.

## 2. The Proposed Algorithm

According to the constrained Bayesian principle [35], the nonlinear discrete system model is considered.

(1) $$x_{k}=f_{k}\left(x_{k-1}\right)+e_{k-1}$$

(2) $$\theta_{k}=h_{\theta_{k}}\left(x_{k}\right)$$

(3) $$\theta_{k}=h_{\theta_{k}}\left(x_{k}\right)$$

where k∈N denotes the discrete time, $f_{k}$, $h_{k}$ and $h_{\theta_{k}}$ denote some appropriate nonlinear functions, and $x_{k}$ is a state vector. $z_{k}$ is a measurement vector. $e_{k}$ is the process noise, with zero mean and covariance $Q_{e_{k}}$, and $v_{k}$ is the measurement noise, with zero mean and covariance $R_{v_{k}}$. $\theta_{k}$ denotes the spatial-temporal feature information.

As is well known, the spatial relationship cannot be directly applied to the particle filtering algorithm. In this paper, the target feature information is used to construct the importance density function through the proposed T-S fuzzy model, so that the spatial relation is introduced into the particle filtering algorithm. In the particle filtering framework, the probability density function estimation is divided into two phases: time update and state update. The prediction state density is calculated from the prior probability density function (PDF) by using the Chapman-Kolmogorov equation:

(4) $$p\left(x_{k}|z_{1:k-1},\theta_{1:k-1}\right)=\int p\left(x_{k}|x_{k-1}\right)p\left(x_{k-1}|z_{1:k-1},\theta_{1:k-1}\right)dx_{k-1}$$

where $p\left(x_{k}|x_{k-1}\right)$ is the priori transfer density function, and the measurement update is computed by a Bayesian formula:

(5) $$p\left(x_{k}|z_{1:k},\theta_{1:k}\right)=\frac{p\left(z_{k}|x_{k}\right)p\left(\theta_{k}|x_{k}\right)p\left(x_{k}|z_{1:k-1},\theta_{1:k-1}\right)}{p\left(z_{k}|z_{1:k-1},\theta_{k}\right)p\left(\theta_{k}|\theta_{1:k-1}\right)}$$

where $p(z_{k}|x_{k})$ is the likelihood function, and $p(\theta_{k}|x_{k})$ is the characteristic likelihood function.

Suppose that ${\left\{x_{k,j}\right\}}_{j=1}^{M}$ represents the particles at time k, where M is the number of particles. Under the constraint of spatial-temporal feature $\theta_{1:k}^{m},\;m=1,\ldots ,G$, and $\sum_{j}^{M}\varpi_{k,j}=1$, $\varpi_{k,j}$ denotes the weight of the particle; consequently,

(6) $$\varpi_{k,j}^{}\propto \varpi_{k-1,j}^{}\frac{p(z_{k}|x_{k,j}^{})p(\theta_{k}|x_{k}^{})p(x_{k,j}^{}|x_{k-1,j}^{})}{q(x_{k,j}^{}|x_{k-1,j}^{},z_{k})}$$

The $p(\theta_{k}|x_{k})$ not only contains abundant prior knowledge, but also incorporates a higher-level spatial-temporal feature as well as measurement information. If we combine it with a prior probability density function $p(x_{k,j}|x_{k-1,j})$ to form an importance density function $q(x_{k,j}|x_{k-1,j},z_{k})$, it will reduce the degradation of the particles.

(7) $$q(x_{k,j}^{}|x_{k-1,j}^{},z_{k})=p(\theta_{k}|x_{k}^{})p(x_{k,j}^{}|x_{k-1,j}^{})$$

### 2.1. Construction of Importance Density Function

**Figure 1:**
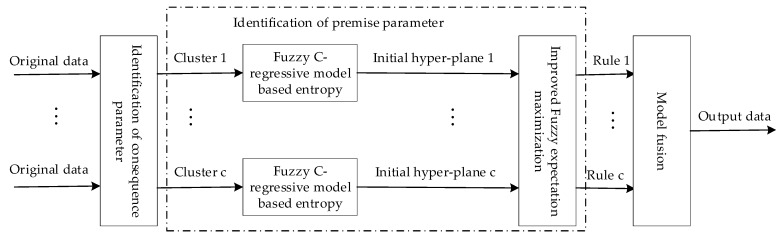
Block diagram of the T-S fuzzy modeling method.

The selection of an importance density function is a very important step. Traditional particle filtering algorithms usually use priori probability as the importance density function, but generally the prior probability does not fully consider the real-time effects of the current measurement. It is easy to cause particle degradation. In order to solve this problem, according to the principle of constrained particle filtering, the importance density function is constructed, as in Equation (7), not only reducing the particle degradation phenomenon, but also improving the stability of the system. The block diagram of the T-S fuzzy modeling method is shown in Figure 1.

#### 2.1.1. T-S Fuzzy Semantic Modeling

A detailed T-S fuzzy model can be found in references [36,37], and it is briefly described in this section. In general, the T-S fuzzy model can be described by $N_{f}$ fuzzy linear models:

Model i: IF $\theta_{k}^{1}$ is $A_{k}^{i,1}$, $\theta_{k}^{2}$ is $A_{k}^{i,2}$, …, $\theta_{k}^{G}$ is $A_{k}^{i,G}$, then:

(8) $$x_{k}^{i}=\Phi_{k-1}^{i}x_{k-1}^{i}+e_{k-1}^{i}$$

(9) $$z_{k}^{i}=H_{k}^{i}x_{k}^{i}+v_{k}^{i},\;i=1,2,\ldots ,N_{f}$$

where $\theta_{k}=\left[\theta_{k}^{1},\theta_{k}^{2},\ldots ,\theta_{k}^{G}\right]$ denotes the premise parameters of the model, $A_{k}^{i,G}$ denotes the fuzzy set of the Gth premise parameter in the model i, and $\Phi_{k-1}^{i}$ and $H_{k}^{i}$ denote the state transition matrix and the measurement matrix, respectively. The consequent part is iteratively updated by the strong tracking algorithm [38], so the global fuzzy model can be represented as follows:

(10) $$x_{k}=\sum_{i=1}^{N_{f}}\mu_{k}^{i}(\theta_{k})(\Phi_{k-1}^{i}x_{k-1}^{i}+e_{k-1}^{i})$$

(11) $$z_{k}=\sum_{i=1}^{N_{f}}\mu_{k}^{i}(\theta_{k})H_{k}^{i}x_{k}^{i}+v_{k}$$

where $\mu_{k}^{i}\left(\theta_{k}\right)$ denotes the model probability, which is calculated as follows:

(12) $$\mu_{k}^{i}(\theta_{k})=\frac{{\bar{\mu }}_{k}^{i}(\theta_{k})}{\sum_{j=1}^{N_{f}}{\bar{\mu }}_{k}^{j}(\theta_{k})},{\bar{\mu }}_{k}^{i}(\theta_{k})=\prod_{m=1}^{G}p_{A_{k}^{i,m}}(\theta_{k}^{m})$$

were $p_{A_{k}^{i,m}}\left(\theta_{k}^{m}\right)\in \left[0,1\right]$ denotes the membership function of the premise parameter $\theta_{k}^{m}$ belonging to model set $A_{k}^{i,m}$ in the fuzzy linear model i.

In general, the fuzzy membership function of the model sets $A_{k}^{i,m}$ is designed as the following Gaussian type function:

(13) $$p_{A_{k}^{i,m}}(\theta_{k}^{m})=\exp\left\{-\frac{1}{2}{\left(\frac{\theta_{k}^{m}-\tau_{k}^{i,m}}{\sigma_{k}^{i,m}}\right)}^{2}\right\}$$

where $\tau_{k}^{i,m}$ and $\sigma_{k}^{i,m}$ denote the mean and standard deviation of the membership function of the premise parameter m in the model i, respectively.

#### 2.1.2. Premise Parameter Identification Based on Improved Fuzzy Expectation Maximization

Inspired by the Gaussian mixed model (GMM) [39], in which the expectation maximization (EM) algorithm was used to fit its parameters, in the process of constructing the T-S model we use the EM algorithm, a general method for a maximum likelihood estimation of the model parameters, to identify the premise parameters. According to the idea of the EM in the GMM, the likelihood function of the parameter model is constructed as follows:

(14) $$L(\theta_{k})=\sum_{l=1}^{C}\log\sum_{i=1}^{N_{f}}\pi_{k}^{i}p_{A_{k}^{i,m}}(\theta_{k}^{m})$$

where $\pi_{k}^{i}$ is a hidden feature, and $p_{A_{k}^{i,m}}\left(\theta_{k}^{m}\right)$ is defined in (13).

Given the appropriate initial assumptions, the noise samples and normal samples participate in the iterative process equally, which will undoubtedly have a negative impact on the accuracy and convergence rate of the parameter estimation. To solve this problem, the knowledge of the fuzzy theory was introduced in the iterative process of the EM algorithm, and a fuzzy expectation maximization (FEM) algorithm [40] was proposed. The fuzzy theory was introduced into the EM algorithm to reduce the influence of noise by making different samples play different roles in the iterative process. Simulations showed that this limitation can better realize the parameter estimation function of the EM algorithm and accelerate the convergence speed of the algorithm.

**Theorem 1**  ***(Jensen inequality [41]):** let* f(x) *be a convex function, for the random variable* X *, then*

f(EX)≥E[f(X)]

*If and only if* X=EX *(That is,* X *is constant), equal sign holds by probability 1, where* E[⋅] *is the expectation operation. For example, the logarithmic function is an upper convex function, then*

log(EX)≥E[log(X)]

In accordance with the traditional FEM, the non-negative fuzzy parameter $u_{k,l}^{i}$ is introduced into Equation (14), where $u_{k,l}^{i}$ is the fuzzy membership degree between the *l*th measurement and the *i*th rule at time k, which satisfies $\sum_{i=1}^{N_{f}}u_{k,l}^{i}=1,\;k=1,\;2,\cdots ,N$, and the Jensen inequality (Theorem 1) is used to obtain the likelihood function of the proposed FEM approach:

(15) $$\begin{array}{ll} L(\tau_{k}^{i,m},\sigma_{k}^{i,m}) & =\sum_{l=1}^{C}\log\sum_{i=1}^{N_{f}}u_{k,l}^{i}\frac{\pi_{k}^{i}p_{A_{k}^{i,m}}(\theta_{k}^{m})}{u_{k,l}^{i}}\\ & =\sum_{l=1}^{C}\log E_{u_{k,l}^{i}}^{}\frac{\pi_{k}^{i}p_{A_{k}^{i,m}}(\theta_{k}^{m})}{u_{k,l}^{i}}\geq \sum_{l=1}^{C}E_{u_{k,l}^{i}}^{}\left[\log\frac{\pi_{k}^{i}p_{A_{k}^{i,m}}(\theta_{k}^{m})}{u_{k,l}^{i}}\right] \end{array}$$

In the EM algorithm, the right side of the inequality in Equation (15) is the lower bound that needs to be optimized:

(16) $$B(\tau_{k}^{i,m},\sigma_{k}^{i,m})=\sum_{l=1}^{C}E_{u_{k,l}^{i}}^{}\left[\log\frac{\pi_{k}^{i}p_{A_{k}^{i,m}}(\theta_{k}^{m})}{u_{k,l}^{i}}\right]=\sum_{l=1}^{C}\sum_{i=1}^{N_{f}}u_{k,l}^{i}\log\frac{\pi_{k}^{i}p_{A_{k}^{i,m}}(\theta_{k}^{m})}{u_{k,l}^{i}}$$

Consider the constraint $\sum_{i=1}^{N_{f}}u_{k,l}^{i}=1,\;\sum_{i=1}^{N_{f}}\pi_{k}^{i}=1$, and introduce the Lagrangian term to obtain the constrained optimization likelihood objective function:

(17) $$\begin{array}{ll} B(\tau_{k}^{i,m},\sigma_{k}^{i,m}) & =\sum_{l=1}^{C}\sum_{i=1}^{N_{f}}u_{k,l}^{i}\log\pi_{k}^{i}+\sum_{l=1}^{C}\sum_{i=1}^{N_{f}}u_{k,l}^{i}\log p_{A_{k}^{i,m}}(\theta_{k}^{m})-\sum_{l=1}^{C}\sum_{i=1}^{N_{f}}u_{k,l}^{i}\log u_{k,l}^{i}\\ & -\lambda_{1}\left(\sum_{i=1}^{N_{f}}\pi_{k}^{i}-1\right)-\lambda_{2}\left(\sum_{i=1}^{N_{f}}u_{k,l}^{i}-1\right) \end{array}$$

It is known from Equation (13) that the membership function of the premise parameter obeys the Gaussian distribution.

(18) $$\begin{array}{ll} B(\tau_{k}^{i,m},\sigma_{k}^{i,m}) & =-\frac{1}{2}\sum_{l=1}^{C}\sum_{i=1}^{N_{f}}u_{k,l}^{i}\log\left({\left(2\pi \right)}^{d}\left|\sigma_{k}^{i,m}\right|\right)-\frac{1}{2}\sum_{l=1}^{C}\sum_{i=1}^{N_{f}}u_{k,l}^{i}{\left(\theta_{k}^{m}-\tau_{k}^{i,m}\right)}^{T}{\left(\sigma_{k}^{i,m}\right)}^{-1}\left(\theta_{k}^{m}-\tau_{k}^{i,m}\right)\\ & +\sum_{l=1}^{C}\sum_{i=1}^{N_{f}}u_{k,l}^{i}\log\pi_{k}^{i}-\sum_{l=1}^{C}\sum_{i=1}^{N_{f}}u_{k,l}^{i}\log u_{k,l}^{i}-\lambda_{1}\left(\sum_{i=1}^{N_{f}}\pi_{k}^{i}-1\right)-\lambda_{2}\left(\sum_{i=1}^{N_{f}}u_{k,l}^{i}-1\right) \end{array}$$

According to the gradient descent method, the mean and standard deviation of the premise membership functions are obtained (The specific derivation process is shown in Appendix A).

(19) $$\tau_{k}^{i,m}=\frac{\sum_{l=1}^{C}u_{k,l}^{i}\theta_{k}^{m}}{\sum_{l=1}^{C}u_{k,l}^{i}}$$

(20) $$\sigma_{k}^{i,m}=\frac{\sum_{l=1}^{C}u_{k,l}^{i}\left(\theta_{k}^{m}-\tau_{k}^{i,m}\right){\left(\theta_{k}^{m}-\tau_{k}^{i,m}\right)}^{T}}{\sum_{l=1}^{C}u_{k,l}^{i}}$$

It can be seen from Equations (19) and (20) that the identification of the premise parameters is closely related to the non-negative fuzzy parameters $u_{k,l}^{i}$. Therefore, the design of the $u_{k,l}^{i}$ is the key problem to be solved in the next step. In the traditional method, the fuzzy parameter was set by manual initialization, which will have certain errors and subjective factors. To avoid the influence, we use the fuzzy C-recessive model (FCRM) clustering algorithm, based on spatial-temporal information and entropy adjustment to obtain the fuzzy parameter. Therefore, the premise parameter membership functions can fully reflect the motion information of the target, and avoid the unnecessary influence caused by the artificial setting of the initial value. Suppose that $z_{k}={\left\{z_{k,l}\right\}}_{l=1}^{C}$ is a measurement set and ${\hat{z}}_{k}={\left\{{\hat{z}}_{k}^{i}\right\}}_{i=1}^{N_{f}}$ is a predictive measurement set, $z_{k,l}$ denotes the *l*th measurement, and ${\hat{z}}_{k}^{i}$ denotes the predictive measurement based on the *i*th fuzzy rule at time k. On the basis of the traditional FCRM [33], the weighted entropy is introduced to balance the membership degree, which is called the entropy adjustment method. Meanwhile, the target feature information reflects the target motion trend in real time. Therefore, combining the spatial constraint information $\theta_{k}$, the objective function of the entropy adjustment method is defined as follows:

(21) $$Y\left(u_{k,l}^{i}\right)=-\sum_{l=1}^{C}\sum_{i=1}^{N_{f}}u_{k,l}^{i}\ln\left(u_{k,l}^{i}\right)-\beta \cdot \sum_{l=1}^{C}\sum_{i=1}^{N_{f}}u_{k,l}^{i}\left[{\left(D_{k,l}^{i}\right)}^{2}+\sum_{m=1}^{G}\omega_{k}^{i,m}\theta_{k}^{m}\right]+\sum_{l=1}^{C}\lambda_{k}\left(\sum_{i=1}^{N_{f}}u_{k,l}^{i}-1\right)$$

where $\lambda_{k}$ is the Lagrange multiplier vector, β is a constant, $\omega_{k}^{i,m}$ is the weight of the feature m in the model i. ${\left(D_{k,l}^{i}\right)}^{2}$ denotes the dissimilarity measure function between the *l*th measurement and the output predictive measurement of the *i*th fuzzy rule, which is defined as:

(22) $${\left(D_{k,l}^{i}\right)}^{2}=\frac{1}{p_{i}(z_{k,l}|{\hat{x}}_{k}^{i})}$$

where $p\left(z_{k,l}|{\hat{x}}_{k}^{i}\right)$ is called the likelihood function of the measurement $z_{k,l}$ given the target state ${\hat{x}}_{k}^{i}$.

According to the Lagrangian multiplier, the update of the fuzzy membership degree $u_{k,l}^{i}$ between the *l*th measurement and the *i*th fuzzy model is obtained (The specific derivation process is shown in Appendix B).

(23) $$u_{k,l}^{i}=\frac{\exp\left(-\beta \left[{\left(D_{k,l}^{i}\right)}^{2}+\sum_{m=1}^{G}\omega_{k}^{i,m}\theta_{k}^{m}\right]\right)}{\sum_{q=1}^{N_{f}}\left(\exp\left(-\beta \left[{\left(D_{k,l}^{q}\right)}^{2}+\sum_{m=1}^{G}\omega_{k}^{q,m}\theta_{k}^{m}\right]\right)\right)}$$

The improved fuzzy expectation maximization algorithm used in the identification of the premise parameters is shown in Algorithm 1.


**Algorithm 1 Premise Parameter Identification-Improved Fuzzy Expectation Maximization**
1. **Initializations:** Define the initial premise parameter ${\left(\tau ,\sigma \right)}^{\left(0\right)}$, the stop criterion ζ, the parametric model likelihood function L(θ), and the set i=0. 2. **Do**
i=i+1Calculate the non-negative fuzzy membership degree by Equation (23) and introduce it into the likelihood function L(θ) of Equation (14), and rewrite the likelihood function into Equation (15).The lower bound function B(θ) is obtained from Jensen’s inequality.E-step: Calculate $B\left(\left(\tau ,\sigma \right),{\left(\tau ,\sigma \right)}^{\left(i-1\right)}\right)$ by Equations (16)–(18).M-step: ${\left(\tau ,\sigma \right)}^{\left(i\right)}=\arg\max B\left(\left(\tau ,\sigma \right),{\left(\tau ,\sigma \right)}^{\left(i-1\right)}\right)$, and the ${\left(\tau ,\sigma \right)}^{\left(i\right)}$ is obtained by Equations (19) and (20). 3. **Until**
$B\left({\left(\tau ,\sigma \right)}^{\left(i+1\right)},{\left(\tau ,\sigma \right)}^{\left(i\right)}\right)-B\left({\left(\tau ,\sigma \right)}^{\left(i\right)},{\left(\tau ,\sigma \right)}^{\left(i-1\right)}\right)\leq \zeta$ 4. **Return**
$\left(\tau ,\sigma \right)={\left(\tau ,\sigma \right)}^{\left(i+1\right)}$ 5. **Finish**

On the basis of the model fusion method in the traditional multiple model algorithm, the premise membership function is identified by Equations (19) and (20), the model probability is obtained by Equation (12), and the ${\hat{x}}_{k}^{i}$ and $P_{k}^{i}$ are obtained by strong tracking [38]. Therefore, the state and covariance updates of the proposed T-S fuzzy model are as follows:

(24) $${\tilde{x}}_{k}=\sum_{i=1}^{N_{f}}\mu_{k}^{i}{\hat{x}}_{k}^{i}$$

(25) $${\tilde{P}}_{k}=\sum_{i=1}^{N_{f}}\mu_{k}^{i}[P_{k}^{i}+({\tilde{x}}_{k}-{\hat{x}}_{k}^{i}){\left({\tilde{x}}_{k}-{\hat{x}}_{k}^{i}\right)}^{T}]$$

For each particle, the state and covariance are ${\tilde{x}}_{k,j}$ and ${\tilde{P}}_{k,j}$ on the basis of Equations (24) and (25), so the importance density function of the proposed algorithm is defined as:

(26) $$q(x_{k,j}^{}|x_{k-1,j}^{},z_{k})=N({\tilde{x}}_{k,j}^{},{\tilde{P}}_{k,j}^{})$$

### 2.2. Summary of the Algorithm

Based on the above analysis, the T-S fuzzy modeling particle filtering algorithm can be summarized as Algorithm 2:


**Algorithm 2 Fuzzy Expectation Maximization-Based T-S Fuzzy Particle Filtering Algorithm**
1. **Initializations:** Set that the number of fuzzy rules is $N_{f}$. The particles ${\left(x_{0:k-1}^{j}\right)}_{j=1}^{M}$ are drawn from the priori probability density function $p\left(x_{0}\right)$, and the number of particles is set to M. 2. **For**
*k* = 1, 2, …(a)T-S fuzzy model parameter identification▪Consequence parameter identification: The strong tracking algorithm [38] is used to identify the consequence parameters.▪Premise parameter identification: As shown in Algorithm 1.(b)Model probability update and fusion: The model probability is updated by Equation (12), and the model fusion is carried out by Equation (10).(c)Construct the importance density function and sample: Draw particles ${\left\{x_{k,j}\right\}}_{j=1}^{M}$ from Equation (26).(d)Calculate and normalize the particle weight: The particle weight is calculated by Equation (6) and normalized as follows:$$\psi_{k,j}^{}=\varpi_{k,j}/\sum_{j=1}^{M}\varpi_{k,j}$$(e)State and covariance estimation:▪Output state: ${\hat{x}}_{k}=\sum_{j=1}^{M}\psi_{k,j}x_{k,j}$▪Output covariance: $P_{k}=\sum_{j=1}^{M}\psi_{k,j}[{\tilde{P}}_{k,j}+\left({\hat{x}}_{k}-x_{k,j}\right)\left({\hat{x}}_{k}-x_{k,j}{)}^{T}\right]$

### 2.3. Discussion

Summary: In the design of the proposed algorithm, to improve the convergence performance of the T-S fuzzy model in a passive sensor system, and to reduce the approximation error, the FEM was introduced to identify the premise parameters that can capture the higher-order statistical parameters from a small number of samples. To identify the consequent parameters of the T-S fuzzy model, the strong tracking estimator was used. In particular, for the maneuvering target tracking, the model probability, which was closely connected to the true motion model, was adaptively updated by the premise membership functions. Moreover, the samples were drawn from the proposed T-S fuzzy model, which had abundant priori information and the latest measurement, and which can reduce the degradation of the particles.

Comparison: All of the samples were used to train the fuzzy model parameters in the conventional T-S fuzzy model described in [33,34], after which the trained fuzzy model was used to classify or estimate the model state. In our proposed algorithm, the parameters of the T-S fuzzy model were updated by using the recursive mechanism of the algorithm, which required the fast convergence. The simulations showed that the proposed algorithm can not only achieve fast convergence, but that it can also accurately estimate the state.

Additionally, in the traditional FEM, the fuzzy parameter was set by manual initialization, but in our proposed algorithm the fuzzy parameter was obtained through the FCRM, based on entropy and the spatial-temporal characteristic information. The simulations showed that not only can the proposed FEM realize the parameter estimation function of the EM algorithm and accelerate the convergence speed, but it can also avoid the subjective influence caused by the artificial setting of the initial value.

## 3. Simulation Results and Analysis

In order to compare the performance of the FEMTS-PF with those of IMMUKF, IMMEKF, IMMRBPF [42], fuzzy PF (FPF) and traditional FEMTS-PF, two examples are employed in this section. In Section 3.1., a bearings-only tracking example is adopted. In Section 3.2., the tracking results of a maneuvering target in a sparse environment are analyzed. 100 Monte Carlo simulations are carried out in all of the experiments, and the number of particles is 200. The proposed algorithm is implemented on Matlab R2017a on the CPU3.6 and 8 GB memory computers of Inter (R) Core (TM) i5-6500.

The root-mean square error (RMSE) is used as the performance index (PI), which is defined as:

(27) $$RMSE=\sqrt{\frac{\sum_{k=1}^{D}{\left(x_{k}-{\hat{x}}_{k}\right)}^{2}}{D}}$$

where D is the number of Monte Carlo (MC) simulations.

### 3.1. An Example of Bearings-Only Tracking (BOT)

The state and measurement equations of the target in the proposed algorithm are as follows.

(28) $$x_{k}^{i}=\Phi_{k-1}^{i}(T,\omega_{i})x_{k-1}^{i}+e_{k-1}$$

(29) $$z_{k}^{i}=H_{k}^{i}x_{k}^{i}+v_{k},\;i=1,2,\ldots ,N_{f}$$

where $N_{f}$ denotes the number of fuzzy rules, $x_{k}={\left[x_{k},x_{k}^{\prime },y_{k},y_{k}^{\prime }\right]}^{T}$ denotes the state vector, $x_{k}$ denotes the *x*-coordinate and $y_{k}$ denotes the *y*-coordinate of the target. $x_{k}^{\prime }$ and $y_{k}^{\prime }$ denote the corresponding velocities. The process noise $e_{k}$ is assumed to be Gaussian noise with zero mean and standard deviation $\sigma_{i,e}$ (The values are shown in Table 1). The measurement noise $v_{k}$ is assumed to be non-Gaussian noise $\left(R={\left[\frac{N\left(0,\sigma \right)+N\left(0,\sigma \right)}{2},\frac{N\left(0,\sigma \right)+N\left(0,\sigma \right)}{2}\right]}^{T},\sigma =0.001\right)$. The initial state describes the target’s initial position and velocity. The a priori probability density of the state $x_{0}$ is assumed to be $x_{0}\sim N\left({\hat{x}}_{0|0},{\hat{P}}_{0|0}\right)$, where $x_{0|0}=[1\;\mathrm{km},0.15\;\mathrm{km}/s,6\;\mathrm{km},0.26\;\mathrm{km}/s{]}^{T},\;\;P_{0|0}=[\sigma^{2}\;0\;0\;0;0\;\sigma^{2}\;0\;0;0\;0\;\sigma^{2}\;0;0\;0\;0\;\sigma^{2}]$. In this paper, the innovation and the heading angle difference are selected as the semantic information, which can effectively reflect the moving state of the target. For example, the innovation can indicate the suitability of the target motion model; when it is large, the target motion model is explained to be less consistent with the current motion state, and thus we can adjust the model probability to get a more accurate model of motion. The state transition matrix $\Phi_{k-1}^{i}$ is defined as follows:

$$\Phi_{k-1}^{i}=\left[\begin{array}{cccc} 1 & \frac{\sin\omega_{i}T}{\omega_{i}} & 0 & -\frac{1-\cos\omega_{i}T}{\omega_{i}}\\ 0 & \cos\omega_{i}T & 0 & -\sin\omega_{i}T\\ 0 & \frac{1-\cos\omega_{i}T}{\omega_{i}} & 1 & \frac{\sin\omega_{i}T}{\omega_{i}}\\ 0 & \sin\omega_{i}T & 0 & \cos\omega_{i}T \end{array}\right]$$

Table 1 shows the turning rate $\omega_{i}$ and standard deviation $\sigma_{i,e}$ of the process noise in the T-S fuzzy models; $\omega_{i}=0$ denotes the constant turn (CT) model. The process noise covariance is as follows:

$$Q_{e_{k-1}^{i}}=\left[\begin{array}{cccc} \frac{2(\omega_{i}T-\sin\omega_{i}T)}{\omega_{i}^{3}} & \frac{1-\cos\omega_{i}T}{\omega_{i}^{2}} & 0 & \frac{\omega_{i}T-\cos\omega_{i}T}{\omega_{i}^{2}}\\ \frac{1-\cos\omega_{i}T}{\omega_{i}^{2}} & T & \frac{\omega_{i}T-\cos\omega_{i}T}{\omega_{i}^{2}} & 0\\ 0 & -\frac{\omega_{i}T-\cos\omega_{i}T}{\omega_{i}^{2}} & \frac{2(\omega_{i}T-\sin\omega_{i}T)}{\omega_{i}^{3}} & \frac{1-\cos\omega_{i}T}{\omega_{i}^{2}}\\ \frac{\omega_{i}T-\cos\omega_{i}T}{\omega_{i}^{2}} & 0 & \frac{1-\cos\omega_{i}T}{\omega_{i}^{2}} & T \end{array}\right]\cdot \sigma_{i,e}^{2}$$

Two passive sensors are placed in the (0, 5 km) and (0, −5 km). The measurement function:

$$h\left(x_{k}\right)=\left[\begin{array}{c} \beta_{1}\\ \beta_{2} \end{array}\right]=\left[\begin{array}{c} \arctan\left(\frac{y-s_{1,y}}{x-s_{1,x}}\right)\\ \arctan\left(\frac{y-s_{2,y}}{x-s_{2,x}}\right) \end{array}\right]$$

where $s_{i,x},s_{i,y}$ denote the coordinate value of the sensor i. $\beta_{i},\;i=1,\;2$ denotes the azimuth. Then, the Jacobi matrix for each model is:

$$H_{k}^{i}=\left[\begin{array}{l} \begin{array}{cccc} -\frac{y-s_{1,y}}{{\left(x-s_{1,x}\right)}^{2}+{\left(y-s_{1,y}\right)}^{2}} & 0 & \frac{x-s_{1,x}}{{\left(x-s_{1,x}\right)}^{2}+{\left(y-s_{1,y}\right)}^{2}} & 0 \end{array}\\ \begin{array}{cccc} -\frac{y-s_{2,y}}{{\left(x-s_{2,x}\right)}^{2}+{\left(y-s_{2,y}\right)}^{2}} & 0 & \frac{x-s_{2,x}}{{\left(x-s_{2,x}\right)}^{2}+{\left(y-s_{2,y}\right)}^{2}} & 0 \end{array} \end{array}\right]$$

**Figure 2:**
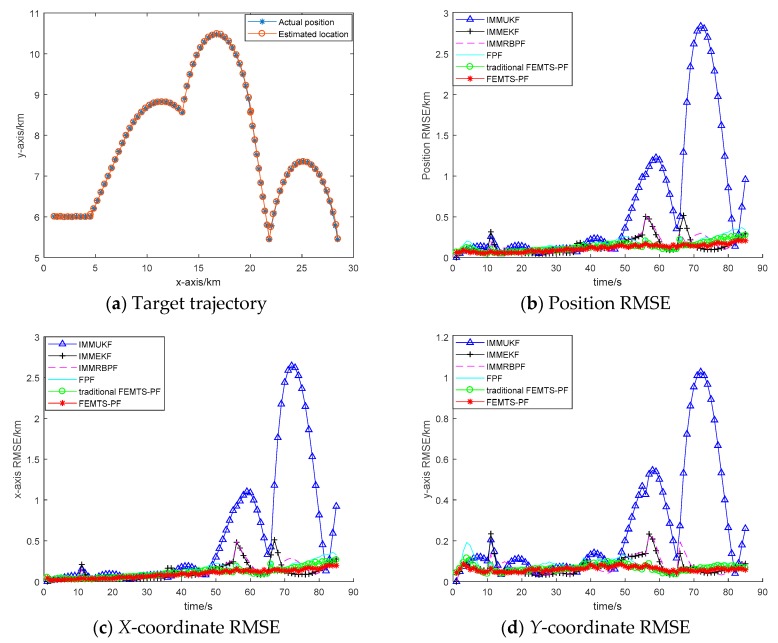
Performance comparison of the IMMUKF (triangle sign (Δ)), IMMEKF (plus sign (+)), IMMRBPF (dotted line (−−)), FPF (solid line (−)), traditional FEMTS-PF (circle (o)) and proposed FEMTS-PF (star sign (∗)). (**a**) The target trajectory of the proposed algorithm and the actual position; (**b**) position root-mean-square error (RMSE); (**c**) *X*-axis RMSE; and (**d**) *Y*-axis RMSE.

In order to verify the effectiveness of the proposed algorithm, the FEMTS-PF is implemented and compared with the IMMUKF, IMMEKF, IMMRBPF, fuzzy particle filtering (FPF) and traditional FEMTS-PF algorithms. Figure 2a denotes the estimated trajectory of the target motion. It can be seen from the diagram that the tracking effect of the FEMTS-PF algorithm is approximately the same as the simulation trajectory; there is no obvious loss of phenomenon. It is shown that the algorithm can deal with uncertain information efficiently in nonlinear systems, while the IMMUKF, IMMEKF and IMMRBPF algorithms have a large error when the target maneuvers; the loss phenomenon of the IMMUKF algorithm is more obvious. The main reason for this is that when the target motion model is uncertain, algorithms such as IMM cannot judge the appropriate model, but the FEMTS-PF algorithm can adaptively adjust the model probability according to the premise membership functions, which results in the posterior probability density constructed by the proposed T-S model being closer to the real posterior probability density function and in the tracking accuracy of the algorithm being improved.

Figure 2b–d describe the RMSE of the position, the *x*-axis and the *y*-axis. The initial mean of innovation (S, L) and heading angle difference (NL, S, PL) in the traditional FEMTS-PF are $m_{k,\nu }^{i}=0,\;2*\sigma \;\mathrm{km}$ and $m_{k,\phi }^{i}=-2,0,2\;\mathrm{rad}$, respectively. In the FPF, there is no precise adjustment to the premise parameter, which is insufficient to meet the moving state of the target. Furthermore, for the traditional FEMTS-PF, the fuzzy parameter of the traditional FEM is set by the empirical value and has a certain subjectivity; thus, it results in a lower precision. It can be seen that the tracking effect of the FEMTS-PF algorithm is better than that of the other algorithms, showing a relatively stable tracking performance. The main reason for this is that the premise parameter of the T-S model is identified by the improved FEM, in which the fuzzy parameter is adaptively updated by the FCRM based entropy and spatial-temporal characteristic information, and it can help to obtain a more accurate T-S model structure. When the motion model is uncertain, by partitioning the state space into several subspaces, and because the weight of the subspaces is updated by the premise parameter membership function, which is described by the characteristic of the target, the global estimation model will be closer to the true motion model. Moreover, since the proposal distribution is chosen using the information from the previous stages, sampling is more efficient; thereby, the particle weights will have a lower variance.

Table 2 shows the statistical results of the RMSE of the proposed FEMTS-PF algorithm for different particle numbers. As can be seen from the table, the position RMSE is decreased with the increase of the number of particles before 200 particles, but the error increases as the number of particles increases after that. The main reason for this is that the sampling variance of the particles is fixed, that is, the distribution range of the particle sets is the same. When the number of particles is too large, the particles are too dense and the phenomenon of particle overlap will appear, which makes its effectiveness decline. If we increase the number of particles as the sampling variance, so that the particle set distribution is loose, the filtering result will be further improved, but this is based on sacrificial computation. Weighing the pros and cons, the number of particles is set to 200.

Table 3 shows the RMSE of the four algorithms with different numbers of particles. It can be seen that when the particle size is 200, the RMSE of the FEMTS-PF algorithm is about 0.1153 km, and the tracking effects are 81.17%, 18.92%, 20.15%, 29.57% and 11.24% higher than the other algorithms, respectively. The results show that the filtering effect of the FEMTS-PF algorithm is more accurate under the same conditions for the reason that the importance density function of the FEMTS-PF is constructed by the proposed T-S fuzzy model, which improves the estimation accuracy.

The running time of a Monte Carlo is shown in Table 4. It can be seen that IMMEKF runs for the least time. As we know, the running time of the particle filtering algorithm is proportional to the number of particles, and the particle resampling step exists in the IMMRBPF algorithm, resulting in a large running time, which improves the estimation efficiency at the expense of the computation. However, in the FEMTS-PF algorithm, the importance density function is constructed by the T-S fuzzy model, which combines the measurement and spatial-temporal information, to effectively improve the diversity of the particles and reduce the degradation of the particles; thus, the resampling step can be omitted. Moreover, the parallel computation is used in the sampling, so the real-time of the FEMTS-PF is largely realized.

**Figure 3:**
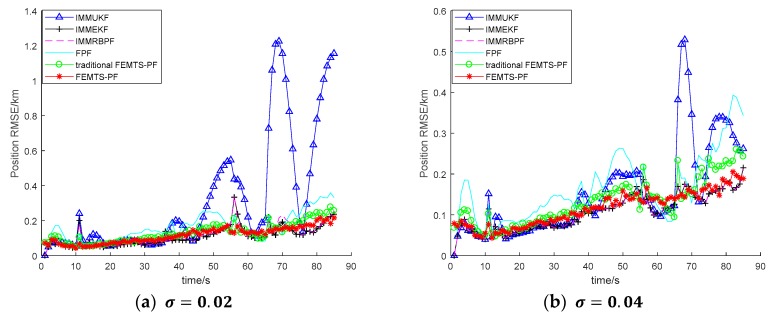
The position RMSE for different process noises. (**a**) The standard deviation of the process noise is 0.02; and (**b**) the standard deviation of the process noise is 0.04.

**Figure 4:**
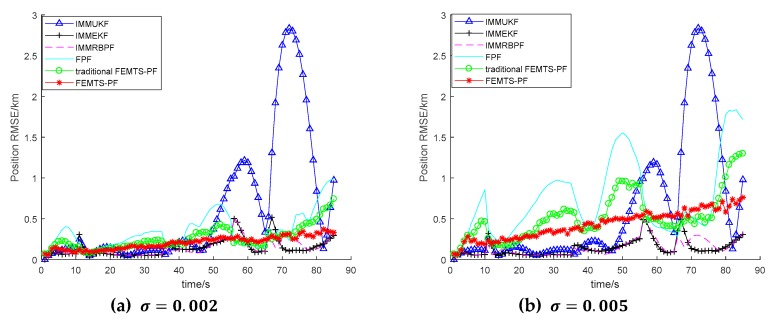
The position RMSE for different measurement noises. (**a**) The standard deviation of the measurement noise is 0.002; and (**b**) the standard deviation of the measurement noise is 0.005.

Figure 3 and Figure 4 are the RMSE of IMMUKF, IMMEKF, IMMRBPF, FPF, traditional FEMTS-PF and FEMTS-PF under different process and observation noises, respectively. The variance of the process noises and observation noises has a great influence on the filtering results. From the view of the signal-to-noise ratio (SNR), the smaller the SNR, the more likely it is that the noise will submerge the real signal, following which the filtering will fail. The larger the SNR, that is, the less noise there is, the easier it is to filter. As can be seen from Figure 3, the greater the noise, the greater the error, which is difficult to avoid. The process noise is reflected in the trust value of the model; the larger the value is, the closer the filtered value is to the measurement value, and a large amount of noise is introduced; the measurement value is a carried error compared with the real value. However, the purpose of filtering is to reduce the noise interference and make the filtering result close to the real value, and if the value of the filtered value and the real value errors are larger, the filtering accuracy is reduced.

As can be seen from Figure 4, the tracking effect of the algorithm decreases with the increase of the noise standard deviation. The larger the measurement noise, the slower the convergence rate of the filter output. On the whole, the FEMTS-PF algorithm is relatively good in dealing with non-Gaussian noise, which proves that the proposed algorithm can effectively solve the nonlinear non-Gaussian problem in the sophisticated system.

### 3.2. Maneuvering Target Tracking in Sparse Environment (SMTT)

In order to further verify the effectiveness of the proposed algorithm, we implement the proposed FEMTS-PF in a sparse environment. The simulation results are compared with those of the IMMUKF, IMMEKF, IMMRBPF and traditional FEMTS-PF algorithms. For the SMTT, the data includes 40 non-periodic sampling points with a target flight time of 107 s, which includes the time interval, *x*-coordinate, *y*-coordinate and batch number of the target. The initial data is $x_{0}=\left[13\;s,\;6.331\;\mathrm{km},\;2.589\;\mathrm{km},\;80\right]$. The sample interval T is not a constant and is defined as follows:

T=t(k+1)−t(k)

where t(k+1) and t(k) are the time interval of k+1 and k, respectively.

**Figure 5:**
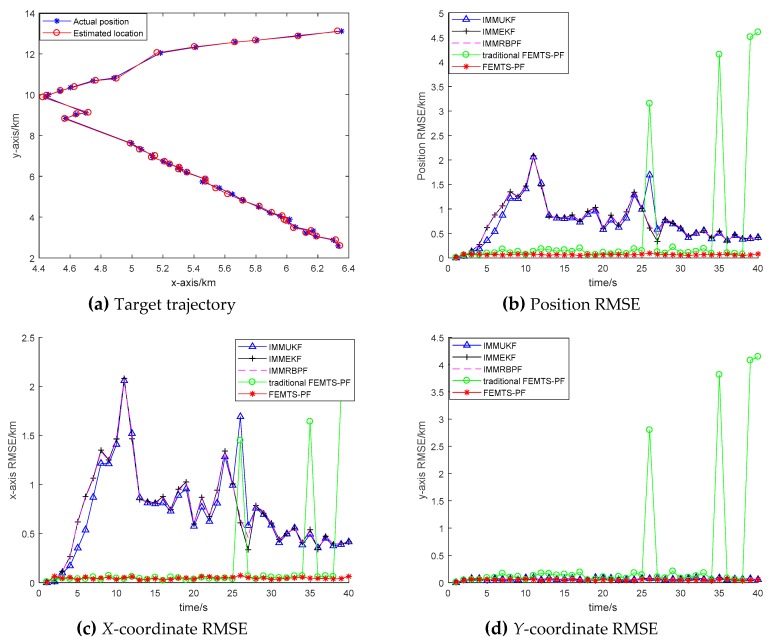
Performance comparison of the IMMUKF (triangle sign (Δ)), IMMEKF (plus sign (+)), IMMRBPF (dotted line (−−)), traditional FEMTS-PF (circle (o)) and FEMTS-PF (star sign (*)). (**a**) The target trajectory of the proposed algorithm and the actual position; (**b**) position root-mean-square error (RMSE); (**c**) *X*-axis RMSE; and (**d**) *Y*-axis RMSE.

Figure 5a denotes the estimated trajectory of the target motion. It can be seen from the diagram that the algorithm is robust while tracking the sparse and more maneuvered radar data. Figure 5b–d describe the RMSE of the position, the *x*-axis and the *y*-axis, respectively. It is shown that the tracking accuracy of the proposed FEMTS-PF algorithm is greatly improved. The position RMSE of the IMMUKF, IMMEKF, IMMRBPF, traditional FEMTS-PF and proposed FEMTS-PF are 0.1010, 0.0998, 0.0979, 0.0991 and 0.0688 km, respectively. The tracking effect of the proposed FEMTS-PF is 31.88%, 31.06%, 29.72% and 30.58% higher than the other algorithms.

The run-time of a Monte Carlo is shown in Table 5. It can be seen that IMMEKF and IMMUKF have a great advantage in real-time performance, but compared with IMMRBPF, the operation time of the FEMTS-PF algorithm is also decreased by 70.80%. Because of the addition of the FCRM algorithm, the proposed FEMTS-PF has a longer operation time than the traditional FEMTS-PF algorithm. However, comparing the performance and computation time of all of the algorithms, the proposed FEMTS-PF algorithm has achieved a good trade-off in the sparse nonlinear non-Gaussian environment.

## 4. Conclusions

In this paper, a novel T-S fuzzy modeling particle filtering algorithm is proposed, in which multiple semantic fuzzy sets are used to represent the spatial-temporal characteristic information of the target, and a general framework of the T-S fuzzy model is constructed, in which the model probability is updated by the premise membership functions. The premise parameters of the T-S fuzzy model are identified by an improved fuzzy expectation maximization algorithm, in which the fuzzy parameter is obtained by a FCRM, based on entropy in order to accelerate the convergence speed and avoid the subjective influence caused by the artificial setting of the fixed value. Meanwhile, a strong tracking method is used to identify the consequence parameters. In the particle filter algorithm, the importance density function is constructed by using the proposed fuzzy model, which improves the diversity of the particles, effectively reduces the degradation of the particles, and omits the particle resampling steps. Furthermore, it improves the real-time performance of the algorithm due to the parallel computation. When the target maneuvering or the moving state of the target is uncertain, the T-S fuzzy model adaptively adjusts the optimal moving state by using the fuzzy semantic information of the innovation and heading angle difference. The results of the examples show that the proposed algorithm is more accurate and stable than the IMMUKF, IMMEKF and IMMRBPF algorithms in the passive sensor system with regards to nonlinear and non-Gaussian problems.

## Figures and Tables

**Table 1:** The $\omega_{i}$ and the $\sigma_{i,e}$ for different $\Delta {\hat{\theta }}_{k}$ and $\Delta \nu_{k}$.

$\Delta {\hat{\theta }}_{k}(\mathrm{rad})$	$\Delta \nu_{k}\;(\mathrm{km})$		
	Negative Large (NL)	Small (S)	Positive Large (PL)
**Small (S)**	−0.0324, 0.15	0, 0.015	0.0324, 0.015
**Large (L)**	−0.0124, 0.005	0, 0.015	0.0124, 0.015

**Table 2:** Average RMSE for different samples of FEMTS-PF.

Particles	Position		*x*-Coordinate		*y*-Coordinate	
	Mean (km)	Var (km^2^)	Mean (km)	Var (km^2^)	Mean (km)	Var (km^2^)
**25**	0.1166	0.0019	0.0949	0.0026	0.0616	0.0001
**50**	0.1158	0.0021	0.0947	0.0027	0.0607	0.0001
**100**	0.1187	0.0021	0.0974	0.0028	0.0617	0.0001
**200**	0.1153	0.0018	0.0943	0.0025	0.0605	0.0001
**500**	0.1159	0.0019	0.0943	0.0025	0.0611	0.0001
**1000**	0.1158	0.0019	0.0946	0.0026	0.0606	0.0001

**Table 3:** Statistics of the position RMSE under different particle numbers.

Particles	IMMUKF		IMMEKF		IMMRBPF		FPF		Traditional FEMTS-PF		FEMTS-PF	
	Mean (km)	Var (km^2^)	Mean (km)	Var (km^2^)	Mean (km)	Var (km^2^)	Mean (km)	Var (km^2^)	Mean (km)	Var (km^2^)	Mean (km)	Var (km^2^)
**25**	0.6122	0.6173	0.1422	0.0109	0.1405	0.0022	0.1658	0.0078	0.1296	0.0033	0.1132	0.0019
**50**					0.1401	0.0238	0.1663	0.0065	0.1288	0.0033	0.1158	0.0021
**100**					0.1411	0.0024	0.1644	0.0059	0.13	0.0032	0.1187	0.0021
**200**					0.1444	0.0956	0.1637	0.0066	0.1299	0.0033	0.1153	0.0018
**500**					0.1495	0.002	0.1638	0.0061	0.1297	0.0031	0.1159	0.0019
**1000**					0.1497	0.0023	0.1645	0.0063	0.1294	0.0031	0.1158	0.0019

**Table 4:** Comparison of the computation time for all of the algorithms (s).

Case	IMMUKF	IMMEKF	IMMRBPF	FPF	Traditional FEMTS-PF	FEMTS-PF
**BOT**	0.0573	0.0353	2.8809	0.9275	1.0316	1.0411

**Table 5:** Comparison of the computation time for all of the algorithms (s).

Case	IMMUKF	IMMEKF	IMMRBPF	Traditional FEMTS-PF	FEMTS-PF
**SMTT**	0.0342	0.0245	1.1600	0.2542	0.3387

## Appendix A

The lower bound that needs to be optimized is as follows:

$$\begin{array}{c} B(\tau_{k}^{i,m},\sigma_{k}^{i,m})=-\frac{1}{2}\sum_{l=1}^{C}\sum_{i=1}^{N_{f}}u_{k,l}^{i}\log\left({\left(2\pi \right)}^{d}\left|\sigma_{k}^{i,m}\right|\right)-\frac{1}{2}\sum_{l=1}^{C}\sum_{i=1}^{N_{f}}u_{k,l}^{i}{\left(\theta_{k}^{m}-\tau_{k}^{i,m}\right)}^{T}{\left(\sigma_{k}^{i,m}\right)}^{-1}\left(\theta_{k}^{m}-\tau_{k}^{i,m}\right)\\ +\sum_{l=1}^{C}\sum_{i=1}^{N_{f}}u_{k,l}^{i}\log\pi_{k}^{i}-\sum_{l=1}^{C}\sum_{i=1}^{N_{f}}u_{k,l}^{i}\log u_{k,l}^{i}-\lambda_{1}\left(\sum_{i=1}^{N_{f}}\pi_{k}^{i}-1\right)-\lambda_{2}\left(\sum_{i=1}^{N_{f}}u_{k,l}^{i}-1\right) \end{array}$$

Derive the $\tau_{k}^{i,m}$ and make it 0:

$$\begin{array}{ll} \frac{\partial B}{\partial \tau_{k}^{i,m}} & =\frac{\partial }{\partial \tau_{k}^{i,m}}\left(-\frac{1}{2}\sum_{l=1}^{C}\sum_{i=1}^{N_{f}}u_{k,l}^{i}{\left(\theta_{k}^{m}-\tau_{k}^{i,m}\right)}^{T}{\left(\sigma_{k}^{i,m}\right)}^{-1}\left(\theta_{k}^{m}-\tau_{k}^{i,m}\right)\right)\\ & =\sum_{l=1}^{C}u_{k,l}^{i}{\left(\sigma_{k}^{i,m}\right)}^{-1}\left(\theta_{k}^{m}-\tau_{k}^{i,m}\right)=0 \end{array}$$

and

$$\sum_{l=1}^{C}u_{k,l}^{i}{\left(\sigma_{k}^{i,m}\right)}^{-1}\theta_{k}^{m}=\sum_{l=1}^{C}u_{k,l}^{i}{\left(\sigma_{k}^{i,m}\right)}^{-1}\tau_{k}^{i,m}$$

The $\tau_{k}^{i,m}$ is obtained as:

$$\tau_{k}^{i,m}=\frac{\sum_{l=1}^{C}u_{k,l}^{i}\theta_{k}^{m}}{\sum_{l=1}^{C}u_{k,l}^{i}}$$

Since $\sigma_{k}^{i,m}$ is a matrix variable, the partial property of the matrix variable requires the following properties: $\frac{\partial log\left|C\right|}{\partial C}={\left(C^{T}\right)}^{-1},\frac{\partial }{\partial C}\left[a^{T}C^{-1}b\right]=-{\left(C^{T}\right)}^{-1}ab^{T}{\left(C^{T}\right)}^{-1},{\left(\sigma_{k}^{i,m}\right)}^{T}=\sigma_{k}^{i,m}$.

Therefore, derive the $\sigma_{k}^{i,m}$ and make it 0:

$$\begin{array}{ll} \frac{\partial B}{\partial \sigma_{k}^{i,m}} & =\frac{\partial }{\partial \sigma_{k}^{i,m}}\left(-\frac{1}{2}\sum_{l=1}^{C}\sum_{i=1}^{N_{f}}u_{k,l}^{i}\log\left(\left|\sigma_{k}^{i,m}\right|\right)-\frac{1}{2}\sum_{l=1}^{C}\sum_{i=1}^{N_{f}}u_{k,l}^{i}{\left(\theta_{k}^{m}-\tau_{k}^{i,m}\right)}^{T}{\left(\sigma_{k}^{i,m}\right)}^{-1}\left(\theta_{k}^{m}-\tau_{k}^{i,m}\right)\right)\\ & =-\frac{1}{2}\sum_{l=1}^{C}u_{k,l}^{i}{\left(\sigma_{k}^{i,m}\right)}^{-1}+\frac{1}{2}\sum_{l=1}^{C}u_{k,l}^{i}{\left(\sigma_{k}^{i,m}\right)}^{-1}\left(\theta_{k}^{m}-\tau_{k}^{i,m}\right){\left(\theta_{k}^{m}-\tau_{k}^{i,m}\right)}^{T}{\left(\sigma_{k}^{i,m}\right)}^{-1}=0 \end{array}$$

and

$$\sum_{l=1}^{C}u_{k,l}^{i}{\left(\sigma_{k}^{i,m}\right)}^{-1}=\sum_{l=1}^{C}u_{k,l}^{i}{\left(\sigma_{k}^{i,m}\right)}^{-1}\left(\theta_{k}^{m}-\tau_{k}^{i,m}\right){\left(\theta_{k}^{m}-\tau_{k}^{i,m}\right)}^{T}{\left(\sigma_{k}^{i,m}\right)}^{-1}$$

$$\sigma_{k}^{i,m}\sum_{l=1}^{C}u_{k,l}^{i}{\left(\sigma_{k}^{i,m}\right)}^{-1}\sigma_{k}^{i,m}=\sigma_{k}^{i,m}\sum_{l=1}^{C}u_{k,l}^{i}{\left(\sigma_{k}^{i,m}\right)}^{-1}\left(\theta_{k}^{m}-\tau_{k}^{i,m}\right){\left(\theta_{k}^{m}-\tau_{k}^{i,m}\right)}^{T}{\left(\sigma_{k}^{i,m}\right)}^{-1}\sigma_{k}^{i,m}$$

$$\sigma_{k}^{i,m}\sum_{l=1}^{C}u_{k,l}^{i}=\sum_{l=1}^{C}u_{k,l}^{i}\left(\theta_{k}^{m}-\tau_{k}^{i,m}\right){\left(\theta_{k}^{m}-\tau_{k}^{i,m}\right)}^{T}$$

The $\sigma_{k}^{i,m}$ is obtained as:

$$\sigma_{k}^{i,m}=\frac{\sum_{l=1}^{C}u_{k,l}^{i}\left(\theta_{k}^{m}-\tau_{k}^{i,m}\right){\left(\theta_{k}^{m}-\tau_{k}^{i,m}\right)}^{T}}{\sum_{l=1}^{C}u_{k,l}^{i}}$$

## Appendix B

The objective function of the entropy adjustment-based FCRM method is defined as follows:

$$Y\left(u_{k,l}^{i}\right)=-\sum_{l=1}^{C}\sum_{i=1}^{N_{f}}u_{k,l}^{i}\ln\left(u_{k,l}^{i}\right)-\beta \cdot \sum_{l=1}^{C}\sum_{i=1}^{N_{f}}u_{k,l}^{i}\left[{\left(D_{k,l}^{i}\right)}^{2}+\sum_{m=1}^{G}\omega_{k}^{i,m}\theta_{k}^{m}\right]+\sum_{l=1}^{C}\lambda_{k}\left(\sum_{i=1}^{N_{f}}u_{k,l}^{i}-1\right)$$

Derive the $u_{k,l}^{i}$ and make it 0:

$$\frac{\partial Y}{\partial u_{k,l}^{i}}=-\left(1+\ln u_{k,l}^{i}\right)-\beta \cdot \left[{\left(D_{k,l}^{i}\right)}^{2}+\sum_{m=1}^{G}\omega_{k}^{i,m}\theta_{k}^{m}\right]+\lambda_{k}=0$$

and

$$\ln u_{k,l}^{i}=-1-\beta \cdot \left[{\left(D_{k,l}^{i}\right)}^{2}+\sum_{m=1}^{G}\omega_{k}^{i,m}\theta_{k}^{m}\right]+\lambda_{k}$$

$$\begin{array}{l} u_{k,l}^{i}=\exp\left(-1-\beta \cdot \left[{\left(D_{k,l}^{i}\right)}^{2}+\sum_{m=1}^{G}\omega_{k}^{i,m}\theta_{k}^{m}\right]+\lambda_{k}\right)\\ \begin{array}{cc} & =\exp\left(-1+\lambda_{k}\right)\times \exp\left(-\beta \cdot \left[{\left(D_{k,l}^{i}\right)}^{2}+\sum_{m=1}^{G}\omega_{k}^{i,m}\theta_{k}^{m}\right]\right) \end{array} \end{array}$$

On the basis of $\sum_{i=1}^{N_{f}}u_{k,l}^{i}=1$:

$$\sum_{i=1}^{N_{f}}u_{k,l}^{i}=\sum_{i=1}^{c}\left(\exp\left(-1+\lambda_{k}\right)\times \exp\left(-\beta \cdot \left[{\left(D_{k,l}^{i}\right)}^{2}+\sum_{m=1}^{G}\omega_{k}^{i,m}\theta_{k}^{m}\right]\right)\right)=1$$

$$\exp\left(-1+\lambda_{k}\right)=\frac{1}{\sum_{j=1}^{c}\exp\left(-\beta \cdot \left[{\left(D_{k,l}^{i}\right)}^{2}+\sum_{m=1}^{G}\omega_{k}^{i,m}\theta_{k}^{m}\right]\right)}$$

The $u_{k,l}^{i}$ is obtained as:

$$u_{k,l}^{i}=\frac{\exp\left(-\beta \left[{\left(D_{k,l}^{i}\right)}^{2}+\sum_{m=1}^{G}\omega_{k}^{i,m}\theta_{k}^{m}\right]\right)}{\sum_{q=1}^{N_{f}}\left(\exp\left(-\beta \left[{\left(D_{k,l}^{q}\right)}^{2}+\sum_{m=1}^{G}\omega_{k}^{q,m}\theta_{k}^{m}\right]\right)\right)}$$

## References

[1] Sinopoli B., Schenato L., Franceschetti M., Poolla K., Jordan M.I., Sastry S.S. (2004). Kalman filtering with intermittent observations. IEEE Trans. Autom. Control.

[2] Yu J., Chen L. (2016). From Static to Dynamic Tag Population Estimation: An Extended Kalman Filter Perspective. IEEE Trans. Commun..

[3] Xiao M., Zhang Y., Wang Z., Fu H. (2018). Augmented robust three-stage extended Kalman filter for Mars entry-phase autonomous navigation. Int. J. Syst. Sci..

[4] Locubiche-Serra S., Seco-Granados G., López-Salcedo J.A. (2018). Closed-Form Approximation for the Steady-State Performance of Second-Order Kalman Filters. IEEE Signal Process. Lett..

[5] Julier S., Uhlmann J.K. (1996). A General Method for Approximating Nonlinear Transformations of Probability Distributions.

[6] Ishihara S., Yamakita M. Gain constrained robust UKF for nonlinear systems with parameter uncertainties. Proceedings of the 2016 European Control Conference (ECC).

[7] Gao B., Hu G., Gao S., Zhong Y., Gu C. (2018). Multi-sensor Optimal Data Fusion for INS/GNSS/CNS Integration Based on Unscented Kalman Filter. Int. J. Control Autom. Syst..

[8] Qi J., Sun K., Wang J., Liu H. (2018). Dynamic State Estimation for Multi-Machine Power System by Unscented Kalman Filter With Enhanced Numerical Stability. IEEE Trans. Smart Grid.

[9] Gordon N.J., Salmond D.J., Smith A.F.M. (1993). Novel Approach to Nonlinear/Non-Gaussian Bayesian State Estimation. IEE Proc. F.

[10] Isard M., Blake A. (1998). Condensation—Conditional Density Propagation for Visual Tracking. Int. J. Comput. Vis..

[11] Li Y., Zhuang X., Liu Y. (2014). UPF Tracking Method Based on Color and SIFT Features Adaptive Fusion. Int. J. Signal Process. Image Process. Pattern Recognit..

[12] Abouzahir M., Elouardi A., Bouaziz S., Latif R., Abdelouahed T. An improved Rao-Blackwellized particle filter based-SLAM running on an OMAP embedded architecture. Proceedings of the 2014 Second World Conference on Complex Systems (WCCS).

[13] Ubeda-Medina L., Garcia-Fernandez A., Grajal J. (2017). Adaptive auxiliary particle filter for track-before-detect with multiple targets. IEEE Trans. Aerosp. Electron. Syst..

[14] Garcia R.V., Silva W.R., Pardal P.C., Kuga H.K., Zanardi M.C. (2017). Sequential nonlinear estimation: Regularized particle filter applied to the attitude estimation problem with real data. Comput. Appl. Math..

[15] Jing L., Vadakkepat P. (2010). Interacting MCMC particle filter for tracking maneuvering target. Digit. Signal Process..

[16] Kotecha J.H., Djuric P.M. (2003). Gaussian particle filtering. IEEE Trans. Signal Process..

[17] Kotecha J.H., Djuric P.M. (2003). Gaussian sum particle filtering. IEEE Trans. Signal Process..

[18] Yu M., Gong L., Oh H., Chen W.H., Chambers J. (2018). Multiple Model Ballistic Missile Tracking with State-Dependent Transitions and Gaussian Particle Filtering. IEEE Trans. Aerosp. Electron. Syst..

[19] Dhassi Y., Aarab A. (2018). Visual tracking based on adaptive interacting multiple model particle filter by fusing multiples cues. Multimed. Tools Appl..

[20] Bando T., Shibata T., Doya K., Ishii S. (2006). Switching particle filters for efficient visual tracking. Robot. Auton. Syst..

[21] Meshgi K., Maeda S.I., Oba S., Skibbe H., Li Y.Z., Ishii S. (2016). An occlusion-aware particle filter tracker to handle complex and persistent occlusions. Comput. Vis. Image Underst..

[22] Martino L., Elvira V., Camps-Valls G. (2017). Group Importance Sampling for Particle Filtering and MCMC. Digit. Signal Process..

[23] Dunne D., Kirubarajan T. (2013). Multiple Model Multi-Bernoulli Filters for Manoeuvering Targets. IEEE Trans. Aerosp. Electron. Syst..

[24] Yang F., Zhang W. (2017). Multiple Model Bernoulli Particle Filter for Maneuvering Target Tracking. J. Electron. Inf. Technol..

[25] Chen Z., Qu Y., Xi Z., Bo Y., Liu B. (2017). Efficient Particle Swarm Optimized Particle Filter Based Improved Multiple Model Tracking Algorithm. Comput. Intell..

[26] Urteaga M., Bugallo F., Djuric P.M. Sequential Monte Carlo methods under model uncertainty. Proceedings of the 2016 IEEE Statistical Signal Processing Workshop (SSP).

[27] Martino L., Read J., Elvira V., Louzada F. (2017). Cooperative parallel particle filters for online model selection and applications to urban mobility. Digit. Signal Process..

[28] Carvalho C.M., Johannes M.S., Lopes H.F., Polson N.G. (2010). Particle Learning and Smoothing. Stat. Sci..

[29] Hounek L., Cintula P. (2006). From fuzzy logic to fuzzy mathematics: A methodological manifesto. Fuzzy Sets Syst..

[30] Widynski N., Dubuisson S., Bloch I. (2011). Integration of Fuzzy Spatial Information in Tracking Based on Particle Filtering. IEEE Trans. Syst. Man Cybern. Part B.

[31] Li L., Li C., Cao W., Liu Z.X. (2016). Fuzzy Quadrature Particle Filter for Maneuvering Target Tracking. Int. J. Fuzzy Syst..

[32] Li L., Xie W., Liu Z. (2016). A novel quadrature particle filtering based on fuzzy c-means clustering. Knowl. Based Syst..

[33] Chang C.W., Tao C.W. (2017). A Novel Approach to Implement Takagi-Sugeno Fuzzy Models. IEEE Trans. Cybern..

[34] Xie X., Lin L., Zhong S. (2014). Process Takagi–Sugeno model: A novel approach for handling continuous input and output functions and its application to time series prediction. Knowl. Based Syst..

[35] Papi F., Podt M., Boers Y., Battistello G., Ulmke M. On constraints exploitation for particle filtering based target tracking. Proceedings of the 2012 15th International Conference on Information Fusion.

[36] Li L.-Q., Wang X.-L., Xie W.-X., Liu Z.-X. (2019). A novel recursive T-S fuzzy semantic modeling approach for discrete state-space systems. Neurocomputing.

[37] Wang X., Li L., Xie W. (2019). T-S Fuzzy Multiple Model Target Tracking Algorithm with UKF Parameter Identification. J. Signal Process..

[38] Li J., Zhao R., Chen J., Zhao C., Zhu Y. (2016). Target tracking algorithm based on adaptive strong tracking particle filter. IET Sci. Meas. Technol..

[39] Vila J.P., Schniter P. (2013). Expectation-Maximization Gaussian-Mixture Approximate Message Passing. IEEE Trans. Signal Process..

[40] Prakash R.M., Kumari R.S.S. (2017). Spatial Fuzzy C Means and Expectation Maximization Algorithms with Bias Correction for Segmentation of MR Brain Images. J. Med Syst..

[41] Buckley S.M. (1993). Estimates for Operator Norms on Weighted Spaces and Reverse Jensen Inequalities. Trans. Am. Math. Soc..

[42] Li L., Xie W., Huang J., Huang J. (2009). Multiple Model Rao–Blackwellized Particle Filter for Maneuvering Target Tracking. Int. J. Def. Sci..

